# Dosimetric feasibility of neurovascular bundle-sparing stereotactic body radiotherapy with periprostatic hydrogel spacer for localized prostate cancer to preserve erectile function

**DOI:** 10.1259/bjr.20200433

**Published:** 2021-03-02

**Authors:** Mark E Hwang, Mark Mayeda, Hiram Shaish, Carl D Elliston, Catherine S Spina, Sven Wenske, Israel Deutsch

**Affiliations:** 1 Department of Radiation Oncology, University of Wisconsin Health Cancer Center at ProHealth Care, Waukesha, WI, USA; 2 Department of Radiation Oncology, Columbia University Medical Center, New York, NY, USA; 3 Department of Radiology, Columbia University Medical Center, New York, NY, USA; 4 Department of Urology, Columbia University Medical Center, New York, NY, USA

## Abstract

**Objective:**

We aim to test the hypothesis that neurovascular bundle (NVB) displacement by rectal hydrogel spacer combined with NVB delineation as an organ at risk (OAR) is a feasible method for NVB-sparing stereotactic body radiotherapy.

**Methods:**

Thirty-five men with low- and intermediate-risk prostate cancer who underwent rectal hydrogel spacer placement and pre-, post-spacer prostate MRI studies were treated with prostate SBRT (36.25 Gy in five fractions). A prostate radiologist contoured the NVB on both the pre- and post-spacer T2W MRI sequences that were then registered to the CT simulation scan for NVB-sparing radiation treatment planning. Three SBRT treatment plans were developed for each patient: (1) no NVB sparing, (2) NVB-sparing using pre-spacer MRI, and (3) NVB-sparing using post-spacer MRI. NVB dose constraints include maximum dose 36.25 Gy (100%), V34.4 Gy (95% of dose) <60%, V32Gy <70%, V28Gy <90%.

**Results:**

Rectal hydrogel spacer placement shifted NVB contours an average of 3.1 ± 3.4 mm away from the prostate, resulting in a 10% decrease in NVB V34.4 Gy in non-NVB-sparing plans (*p* < 0.01). NVB-sparing treatment planning reduced the NVB V34.4 by 16% without the spacer (*p* < 0.01) and 25% with spacer (*p* < 0.001). NVB-sparing did not compromise PTV coverage and OAR endpoints.

**Conclusions:**

NVB-sparing SBRT with rectal hydrogel spacer significantly reduces the volume of NVB treated with high-dose radiation. Rectal spacer contributes to this effect through a dosimetrically meaningful displacement of the NVB that may significantly reduce RiED. These results suggest that NVB-sparing SBRT warrants further clinical evaluation.

**Advances in knowledge:**

This is a feasibility study showing that the periprostatic NVBs can be spared high doses of radiation during prostate SBRT using a hydrogel spacer and nerve-sparing treatment planning.

## Introduction

Men undergoing all forms of definitive therapy for prostate cancer experience a decline in erectile function following treatment.^1–3^ In the Monitoring, Surgery or Radiotherapy for Prostate Cancer (PROTECT) randomized trial,^1^ approximately half of men developed radiation-induced erectile dysfunction (RiED).

While our mechanistic understanding of post-radiotherapy rectal and urinary toxicity is well established, that of post-radiotherapy sexual dysfunction is less clear.^4^ The penile bulb is frequently contoured as an avoidance structure for external beam radiotherapy planning. A range of doses was implicated in studies that showed a correlation between penile bulb dose and toxicity.^5–7^ However, radiation to the penile bulb has not definitively been demonstrated as a cause of RiED.^4,5,8–10^ A number of single and multi-institution series reported new onset erectile dysfunction after radiotherapy that was unexplained by dose-volume relationship analyses of the corpus spongiosum (penile bulb), corpora cavernosa, or crura.^9,11^

Conversely, the urological literature has consistently identified damage to peripheral neurovascular bundles (NVB) surrounding the prostate as a contributor to urinary incontinence and erectile dysfunction.^12^ A recent meta-analysis reported improved urinary and erectile function with nerve-sparing versus non-nerve-sparing prostatectomy in men with prostate cancer - without a detriment to disease-specific outcomes for patients with prostate-confined disease.^13,14^ Furthermore, the extent of nerve-sparing surgery has been correlated with significant differences in functional outcomes. One surgical series reported functional preservation in 28% of men undergoing unilateral, and 72% of men undergoing bilateral, nerve-sparing prostatectomies.^15^

The causative relationship between erectile function and NVB health may also be inferred from existing radiation oncology literature.^16,17^ In one analysis, the probabilities of maintaining erectile function after brachytherapy, external beam radiotherapy, nerve-sparing prostatectomy and radical prostatectomy were 0.76, 0.55, 0.25, and 0.13, respectively.^3^ These data suggest an inverse relationship between functional outcomes and larger and more aggressive fields of radiotherapy or surgery.

Applying the concept of nerve-sparing intervention to radiotherapy requires accurate delineation of the at-risk NVB on MRI and fusion between an MRI and the radiotherapy planning CT. Reproducibility and feasibility of this methodology has previously been reported.^18–21^ Two prospective vessel-sparing radiotherapy treatment regimens that used MRI-angiogram to delineate and avoid erectile vasculature reported 2-year erectile preservation rates that were higher than historical controls, in the range of 80–90% at 2–5 years.^22,23^

The recent use of hydrogel spacers placed between the rectum and prostate to reduce rectal toxicity has also been correlated with lower rates of post-IMRT erectile dysfunction.^24^ The precise mechanism for this finding is unclear. One hypothesis includes displacement of the relevant pelvic and prostatic nerve plexii that traverse the lateral rectal surface. These compelling data are hypothesis-generating and merit further evaluation.

Here we test the hypotheses that rectal hydrogel spacer displaces the NVB away from the high-dose radiation delivered to the prostate and when combined with delineation of the NVB as an organ at risk (OAR) on MRI, NVB-sparing stereotactic body radiotherapy (SBRT) is a feasible treatment planning technique that does not compromise target coverage.

## Methods and materials

This retrospective HIPAA compliant study was approved by the Institutional Review Board with waiver of informed consent.

### Patient selection

Men with low- and intermediate-risk prostate cancer (cT1a-T2c, GS <7, PSA <20) treated with stereotactic body radiotherapy (SBRT) to 3625 cGy in five twice-weekly fractions after hydrogel spacer placement were included in this study. We retrospectively identified 50 consecutively treated men from 2015 to 2019 who underwent a prostate MRI both before and after hydrogel spacer placement and had organ-confined cT1-T2 disease on both digital rectal exam and MRI. Of these, 35 had bilateral NVBs that were visible on both pre- and post-spacer MRI as identified by an expert prostate radiologist. Three Cybermark gold fiducial markers (CIVCO Medical Instruments Co., Inc. Kalona, IA) were implanted in the prostate at the time of spacer placement. The gold markers were readily visible as hypointense punctate spots on MRI.

Transrectal prostate biopsy was performed at least four weeks prior to the first MRI. Men who received androgen deprivation therapy (ADT) were not included in this study to avoid the confounding effects of ADT on both tumor and benign prostate tissue as seen on MRI.^25^

Prostate MRI studies were performed on 3.0T MRI unit with a multi-channel external pelvic phased-array coil: Skyra (Siemens Healthcare, Erlangen, Germany), Signa HDxt (GE Healthcare) and Discovery MR750w (GE Healthcare). The protocol included axial T1W, small FOV 3-plane T2W, axial 2 or 3 b-value DWI (b-values included 0, 50, 1000 and 1500), dynamic axial post-contrast gradient echo imaging (temporal resolution, 7 s) and reconstructed ADC map using a mono-exponential fit of the b-50 and b-1000 images. Detailed MRI protocol parameters can be found in Supplementary Material 1 .

Supplementary Material 1.

### Image registration

An expert prostate radiologist contoured the bilateral NVB structures on all T2W MRI sequences. Each NVB was contoured along the cranio-caudal extent of the prostate. All other target and OAR volumes were contoured on the CT simulation scan as described in our previous publication and Hannan et al.^26,27^

Both the pre-hydrogel spacer MRI and post-hydrogel spacer MRI with NVB contours underwent rigid registration with the CT simulation scan by aligning the posterior prostate wall of the three studies. Fiducial markers were additionally utilized to improve precision of the fusion between the two imaging studies, particularly in cases where alignment along the cranial-caudal axis was in question. After imaging registration, NVB contours were limited only to the axial slices on which the PTV contour was also present. If overlap was found between the PTV and NVB structures, two additional PTVs were created: one excluding the pre-spacer NVB volumes (PTV_PRE_) and another excluding the post-spacer NVB volumes (PTV_POST_).

The distance between the geometric center of each NVB and the CTV was recorded.

### Treatment planning

RapidPlan Knowledge Based Planning (Varian Inc., Palo Alto) was used to design three VMAT SBRT plans for each patient: one without NVB sparing, using RTOG 0938 target coverage and OAR constraints alone; and two with additional NVB dose-limiting constraints planned on the pre- and post-spacer NVB contours. During planning, the structures PTV, PTV_PRE,_ and PTV_POST_ were all subject to the same RTOG 0938 target coverage requirements in each respective plan (*i.e.* 95% of each PTV received 100% of the prescription dose). Rectum, bladder, and bowel OAR treatment planning parameters were followed as defined in RTOG 0938 across all NVB-sparing and non-NVB-sparing plans. Rectum, bladder, and bowel OAR contours for all treatment plans were derived from the post-hydrogel CT simulation scan.

Dose constraints for NVB-sparing treatment plans were as follows for each NVB: maximum dose 36.25 Gy, V_34.4_ (95%) <60%, V_32_ <70%, V_28_ <90%. The V32 (88% of prescribed dose) <70% was extrapolated from Cassidy et al that used a NVB constraint of V_70_ (88% of prescribed dose) <70% based on 1.8 Gy fractions.^18^ The V_34.4_ and V_28_ constraints were implemented in our NVB-sparing plans to ensure sharp high-dose fall-off around the NVB.

Priority weighting of NVB sparing objectives in the RapidPlan treatment planning system was optimized by the planner to facilitate maximal NVB sparing without compromising PTV coverage or exceeding dose constraints to the rectum, bladder, and bowel. DVH end points for PTV and OAR volumes were compared using a two-tailed paired *t*-test.

It should be noted that all three treatment plans for each patient were performed on the same CT-simulation scan with hydrogel spacer in place. This was done so that anatomy and doses to the different NVB contours could be evaluated independent of rectal anatomy that would have changed pre- to post-spacer placement.

### Statistical analysis

One-way analysis of variance (ANOVA) was used to evaluate NVB dosimetric means variation in pre- and post-hydrogel spacer imaging studies, with and without NVB-sparing treatment planning parameters. Comparisons between two groups was evaluated with a student t test. Continuous variables were summarized with means and standard deviations and shown to approximate a normal distribution with a Shapiro-Wilk normality test. Analysis was performed in Office Suite Excel 2019 (Microsoft Corporation, Redmond, WA) and SPSS (IBM, Armonk, NY).

## Results

**Figure 1.**
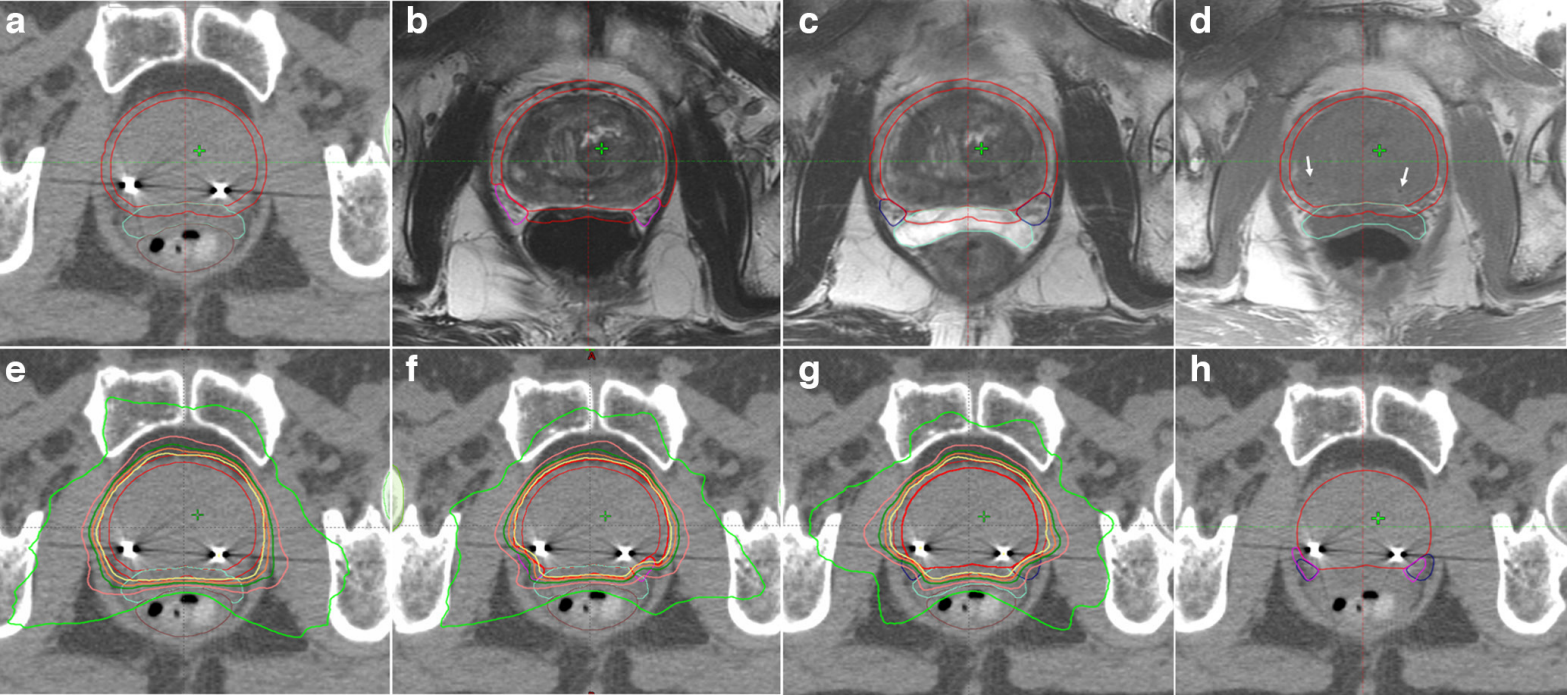
(a) CT axial slice showing CTV, PTV, hydrogel, and rectum contours with two gold fiducial markers. (b) Pre- and (c) post-hydrogel T2W MRI with NVB contours. (d) Post-hydrogel T1W MRI identifying fiducial markers (white arrows). SBRT plan (e) without NVB-sparing, and with NVB-sparing using (f) pre-hydrogel NVB contours and (g) post-hydrogel NVB contours. (h) Pre- and post-hydrogel NVB overlaid on the CT simulation scan. CTV and PTV are in red, pre-hydrogel NVB in magenta and post-hydrogel NVB in blue. Isodose lines in e–g: 100% – yellow, 95% – orange, 90% – dark green, 80% – pink, and 50% – light green.

Thirty-five patients (65 ± 7.2 years) met inclusion criteria and were included in the analysis. Ten men had low-, and 25 had intermediate-risk, prostate cancer. All patients underwent MRI-based pre- and post-hydrogel spacer NVB delineation. There was no difference in NVB volume based on laterality. Pre- and post-hydrogel spacer NVB volumes were 1.64 ± 0.81 cc and 1.71 ± 0.89 cc (mean, standard deviation), respectively. Three treatment plans were created for each patient as described above. A representative axial slice of CT and MRI of the three different plan derivations with isodose lines are shown in Figure 1.

### NVB location

**Figure 2.**
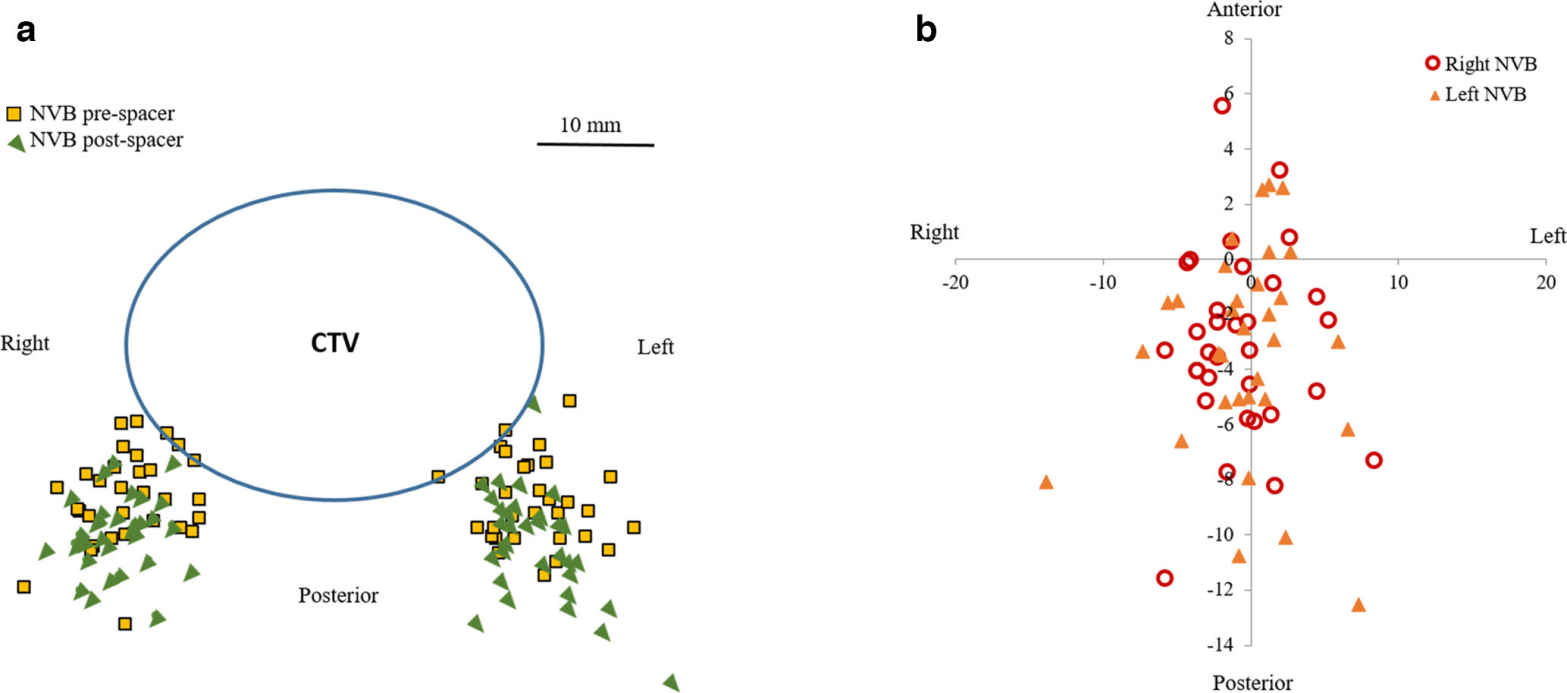
(a) NVB location plotted on prostate mid-gland, axial slice before and after spacer placement. (b) Net NVB translation in mm in axial plane following spacer placement.

The distance between the pre- and post-hydrogel spacer NVB geometric centers relative to the CTV geometric centers on the prostate mid-gland axial slice were measured (Figure 2a). The net NVB translation in the axial plane following spacer placement was 3.1 ± 3.4 mm posteriorly and 0.1 ± 3.2 mm to the left (Figure 2b). No systematic difference in translation of NVB was evident between the left and right NVB structures, nor any pattern of net lateral NVB translation.

### NVB dosimetry

**Figure 3.**
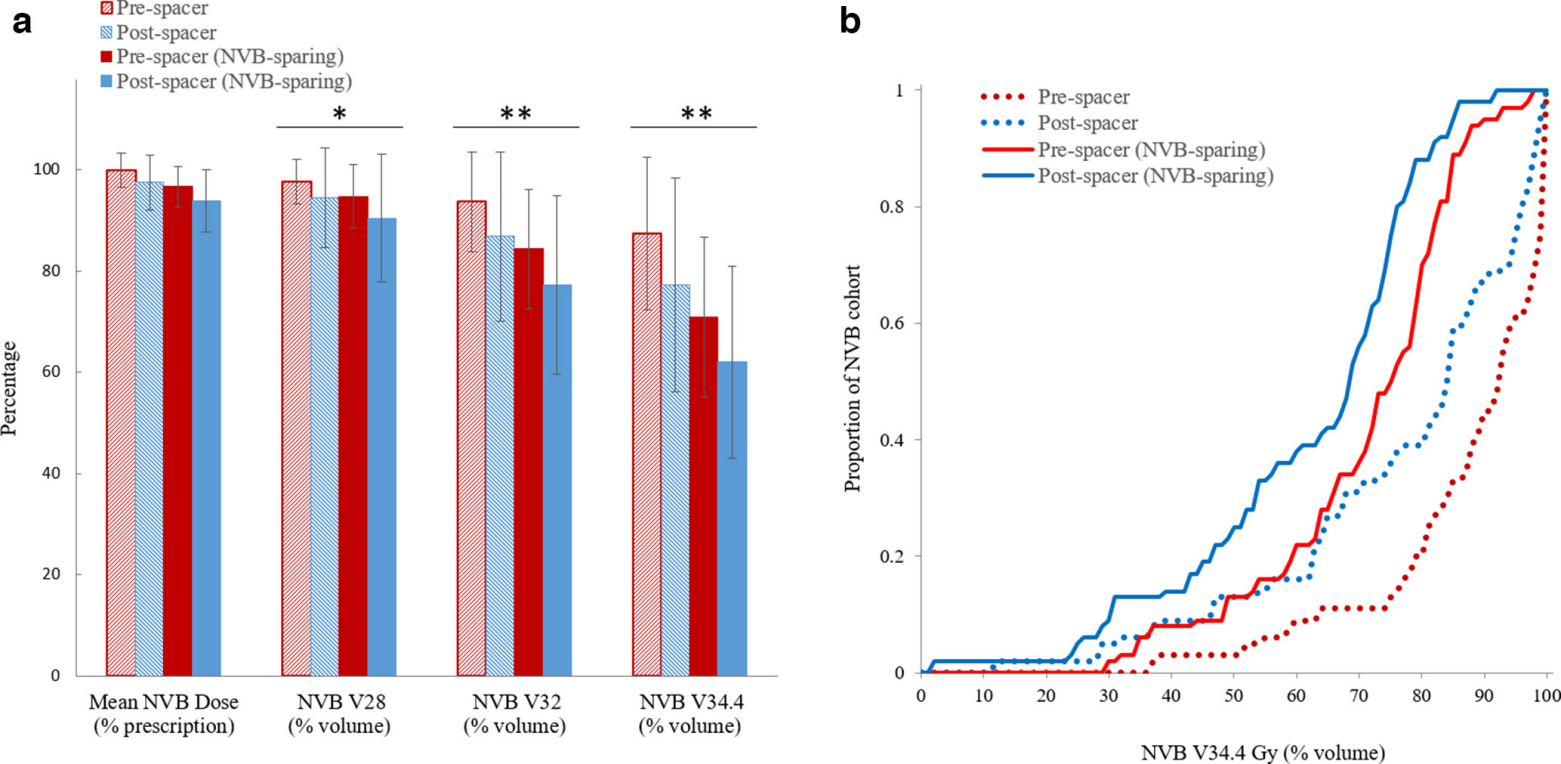
(a) NVB dosimetry mean and standard deviations and (b) cumulative distribution of NVB V34.4 Gy from all NVB contours (*n* = 70), using pre- and post-spacer NVB contours without and with NVB-sparing treatment planning. Statistically significant differences in ANOVA comparison of mean volumes at each NVB dose level in Figure 3a are denoted by *=*p* < 0.01, **=<0.001.

NVB V_28_, V_32_ and V_34.3_, were significantly less after either, or both, hydrogel spacer use and NVB-sparing treatment planning (Figure 3a). The greatest improvement in NVB dosimetry occurred following hydrogel spacer placement and NVB sparing treatment planning. NVB V_28_, V_32_ and V_34.4_ were 98, 93, and 87% in the non-hydrogel spacer plans without NVB sparing, compared with 91, 77, and 62% (*p* < 0.01) in the hydrogel spacer plans with NVB sparing.

Plan evaluation revealed that when neither spacer placement nor NVB-sparing strategies were employed, 10% of NVBs achieved an V34.4 Gy of 70% or less (Figure 3b). The V34.4 <70% increased to approximately 30% with either hydrogel spacer use *or* NVB-sparing planning alone. When the two strategies were combined, the percentage of NVB achieving V34.4 increased to 50%, demonstrating that the combination improved our dosimetric endpoints more than either strategy used alone.

### PTV and OAR dosimetry

**Figure 4.**
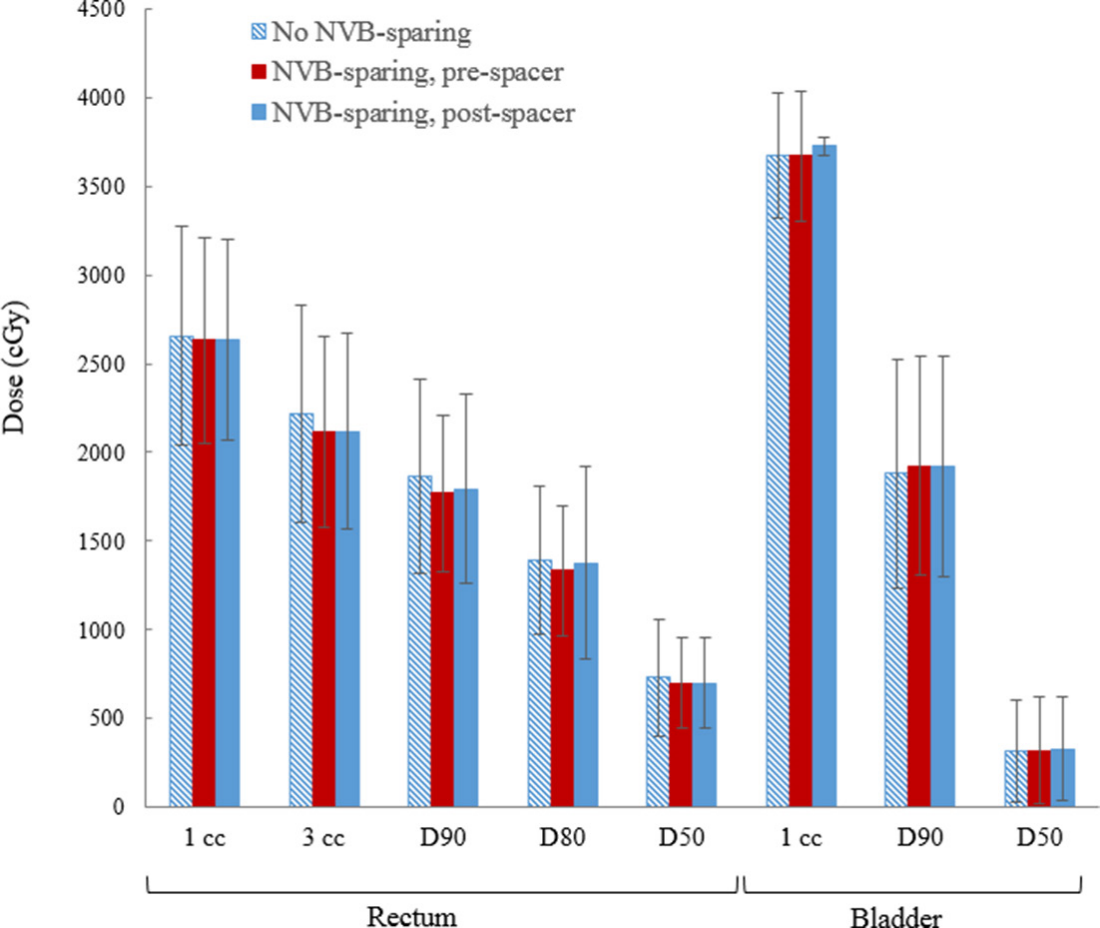
Rectum and bladder organ at risk dosimetry with and without NVB-sparing treatment planning.

PTV coverage and OAR constraints were met for both NVB- and non-NVB-sparing treatment plans (Table 1, Figure 4). A non-significant 1.5% increase in the maximum PTV dose was observed in NVB-sparing treatment plans. Attempts to further spare the NVBs beyond that presented here resulted in additional PTV inhomogeneity and less rectal and bladder sparing.

**Table 1.** PTV mean and standard deviation maximum, D1cc, and D95 doses (as % of prescription dose) without NVB-sparing, and with NVB-sparing using pre-, and post-, spacer NVB contours

	PTV dosimetry comparison					
	PTV without NVB-sparing		PTVpre		PTVpost	
	μ	σ	μ	σ	μ	σ
Max	107.5	1.59	109.11	1.41	109	1.28
D1cc	104.9	1.1	105.9	1.21	105.7	0.99
D95	100.2	1.24	100.2	0.97	100.2	0.82

## Discussion

The results presented herein support the feasibility of NVB-sparing with prostate SBRT following placement of a perirectal hydrogel spacer in an effort to mitigate RiED. To our knowledge, this is the first series to delineate the NVBs before and after hydrogel spacer placement, and show a potentially clinically significant measurable posterior shift in NVBs following hydrogel spacer placement and reduction in dose delivered to NVBs. This study builds on previous work that demonstrated the feasibility of contouring the neurovascular structures in an effort to minimize radiation dose to the NVBs hypothesized to contribute to erectile function^4,18,19^ and others that demonstrated improvement in erectile function after radiotherapy with a hydrogel spacer.^13,24,28^

Decades of clinical data have led investigators to generate hypotheses regarding the etiology of RiED.^5,9,10^ We hypothesize that neurogenic, vasculogenic and psychogenic causes all contribute. Yet, there remains a lack of a mechanistic insight, including characterization of the dose-volume relationships and functional RiED outcomes. Furthermore, the validity of extrapolating the dose-volume relationships and functional outcomes with differing radiation modalities including brachytherapy, 3D conformal RT, IMRT and SBRT has not been proven.

Several recent erectile-function preserving radiation approaches conducted with IMRT have sought to spare the internal pudendal artery (IPA) and the adjacent pelvic nerve plexii in an effort to limit RiED. Dosimetric feasibility of IPA sparing has been demonstrated in conventionally fractionated,^20^ hypofractionated^22^ and SBRT regimens.^29^ Only two single-arm series have published clinical RiED outcomes, as discussed earlier.^22,23^

Sparing neurovascular tissue abutting the prostate (NVB) that are subjected to near-prescription doses has not been clinically evaluated. One might hypothesize that the periprostatic neurovascular tissue will benefit from dose-sparing treatment approach given the proximity to high ablative doses of radiation during SBRT. The ability of modern EBRT treatment techniques (*e.g.* IMRT) to generate conformal dose distributions makes NVB-sparing possible. At the same time, MRI acquired for cancer staging enables accurate delineation of the NVBs. It is noteworthy that several early brachytherapy studies that did not find a relationship between NVB dose and erectile function relied on CT approximation of the NVB rather than true MRI delineation of the structure.^21,30–32^ The applicability of those conclusions to present day EBRT techniques is unclear.

Two dosimetric studies of peri-prostatic NVB-sparing radiotherapy with MRI-guided contours have been published. Cassidy et al., prescribing to 79.2 Gy in 1.8 Gy fractions, successfully reduced V_NVB_70 Gy by nearly 50% and V_NVB_ 60 Gy by 8.5%, at the expense of PTV homogeneity.^18^ Compared with non-NVB sparing plans, the maximum PTV point dose increased by 5 Gy and the V_PTV_105% rose from 6 to 66%. Ciabatti et al. successfully completed NVB-sparing treatment planning using moderately hypofractionated radiotherapy to a prescription dose of 67.5 Gy in 2.7 Gy fractions and showed a more modest reduction in V_NVB_ 60 Gy of 5%.^20^ A wide range of NVB-sparing is thus possible at the cost of PTV-coverage and other OAR-sparing priorities.

The V_NVB_ 32 Gy dose level in our series approximates 88% of the prescription dose, similar to that reported by Cassidy and Ciabatti.^18,20^ The V_NVB_ 32 Gy thus forms a reasonable basis of comparison across the three studies. In the present study, NVB-sparing approaches reduced V_NVB_ 32 Gy by 9% compared with non-NVB sparing plans, independent of hydrogel spacer placement. The V_NVB_ 32 Gy further decreased by 16% with the addition of a perirectal spacer. This improvement is greater still at higher isodose levels: NVB-sparing planning with hydrogel spacer placement reduces V_NVB_ 34.4 Gy by over 25%, while rectal and bladder constraints were met by NVB-sparing radiotherapy plans. The NVB-sparing plans resulted in a non-significant maximum PTV dose increase of 1.5% compared with non-NVB sparing plans.

Karsh et al. theorized that the unexpected improvement in RiED with spacer use seen in the hydrogel spacer randomized controlled trial was due to radiation sparing of erectile neurovascular structures.^13^ Much of the historic data correlating penile bulb dose with RiED resulted in the extrapolation of dose-volume relationships (and hence penile bulb constraints) that are typically defined for doses above EQD2 50 Gy, but not at lower doses discussed by Karsh et al.^5,8,13^

While statistically significant differences in penile bulb exposure to low-radiation doses may play a role in RiED,^6,7^ we posit that the contribution of other neurovascular structures to this toxicity, such as the NVB that is typically subjected to near-prescription doses, should be evaluated. The data presented in this study demonstrate that a posterior shift in NVB position occurs after spacer placement and that leveraging these favorable anatomic changes to explore NVB-related mechanisms of erectile function is feasible.

Another factor that contributes to successful NVB-sparing treatment planning shown here are the smaller PTV expansions and sharper dose fall-off around the target inherent to SBRT techniques.^33–35^ While multiple SBRT series in aggregate suggest similar long-term erectile dysfunction rates between SBRT and other EBRT techniques, reported 2-year RiED rates still range broadly from 25–66%.^34,36,37^ This may be due, in part, to variations in CTV-PTV expansion across series.

Hannan et al.^27^, using a 3 mm CTV to PTV expansion, and Fuller et al.^38^, using 2 mm expansions, documented low 2-year ED rates of 25 and 36%, respectively, after prostate SBRT. Tight margins are not unreasonable given the typical sub-2 mm intrafraction shifts typically observed during such treatments.^39^ The added benefit of such small margins in sparing the adjacent NVB is clear: Liss et al^40^ reported that 28 and 55% of visible NVB fall within 5 and 10 mm radial expansions, respectively, around the prostate.

Limitations of this work are those inherent to dosimetric feasibility and retrospective studies. While clinical outcomes are lacking, we demonstrated that the NVB can be successfully spared high-dose radiation using NVB delineation techniques and placement of rectal hydrogel spacer. Notably, contours of the NVB were performed by a single expert prostate radiologist. Reproducibility was not assessed in this study. Similar NVB contouring techniques, however, have been well-described by Cassidy et al.^18,19^, and a high-degree of reproducibility was demonstrated amongst radiation oncologists who underwent contouring instruction. As noted by previous studies, the generalizability of this NVB-sparing technique is limited to men with visible NVB classically located posterolateral to the prostate, accounting for approximately half to two-thirds of men with prostate cancer.^18,40,41^

Treatment planning was performed using the post-spacer CT simulation scan, with pre- and post-hydrogel spacer NVB contours from the respective T2W MRI sequences fused to the CT. While this approach does not reflect the reality of NVB-sparing treatment planning in spacer-naïve anatomy (*i.e.* pre-spacer rectum and pre-spacer bladder position), it does isolate NVB location as the only altered variable across plans, allowing us to compare the effect of NVB location on NVB sparing planning while maintaining constant the contribution of other OARs to the final treatment plan.

Finally, given inter-fraction shifts in internal anatomy typical of the pelvis, and the close abutment of NVBs with the PTV, day-to-day variation in dose per fraction is more likely to affect the NVB than other organs-at-risk. This variability cannot be evaluated from the treatment plan. Comparison of planned NVB dose with delivered NVB dose will therefore be of clinical interest moving forward.

## Conclusions

NVB-sparing SBRT with hydrogel spacer placement has the potential to significantly reduce the high dose delivered to the NVB. The spacer contributes to this effect by inducing a small but dosimetrically meaningful NVB displacement in the posterior direction. We believe that the described approach to offer clinically meaningful reductions in RiED warrants prospective clinical trials.

## References

[1] Donovan JL, Hamdy FC, Lane JA, Mason M, Metcalfe C, Walsh E, et al. Patient-Reported outcomes after monitoring, surgery, or radiotherapy for prostate cancer. N Engl J Med 2016; 375: 1425–37. doi: 10.1056/NEJMoa1606221 27626365 PMC5134995

[2] Hamdy FC, Donovan JL, Lane JA, Mason M, Metcalfe C, Holding P, et al. 10-Year outcomes after monitoring, surgery, or radiotherapy for localized prostate cancer. N Engl J Med 2016; 375: 1415–24. doi: 10.1056/NEJMoa1606220 27626136

[3] Robinson JW, Moritz S, Fung T. Meta-Analysis of rates of erectile function after treatment of localized prostate carcinoma. Int J Radiat Oncol Biol Phys 2002; 54: 1063–8. doi: 10.1016/S0360-3016(02)03030-4 12419432

[4] Lee JY, Spratt DE, Liss AL, McLaughlin PW. Vessel-sparing radiation and functional anatomy-based preservation for erectile function after prostate radiotherapy. Lancet Oncol 2016; 17: e198–208. doi: 10.1016/S1470-2045(16)00063-2 27301047

[5] Roach M, Nam J, Gagliardi G, El Naqa I, Deasy JO, Marks LB, et al. Radiation dose–volume effects and the penile bulb. Int J Radiat Oncol Biol Phys 2010; 76: S130–4. doi: 10.1016/j.ijrobp.2009.04.094 20171507 PMC4786051

[6] Rasmusson E, Gunnlaugsson A, Wieslander E, Höglund P, Widmark A, Fransson P, et al. Erectile dysfunction and absorbed dose to penile base structures in a randomized trial comparing ultrahypofractionated and conventionally fractionated radiotherapy for prostate cancer. Int J Radiat Oncol Biol Phys 2019; 105: S133–4. doi: 10.1016/j.ijrobp.2019.06.122 32004582

[7] Murray J, Gulliford S, Griffin C, Wilkins A, Syndikus I, Staffurth J, et al. Evaluation of erectile potency and radiation dose to the penile bulb using image guided radiotherapy in the CHHiP trial. Clin Transl Radiat Oncol 2020; 21: 77–84. doi: 10.1016/j.ctro.2019.12.006 32072028 PMC7013161

[8] van der Wielen GJ, Mulhall JP, Incrocci L. Erectile dysfunction after radiotherapy for prostate cancer and radiation dose to the penile structures: a critical review. Radiother Oncol 2007; 84: 107–13. doi: 10.1016/j.radonc.2007.07.018 17707936

[9] Selek U, Cheung R, Lii M, Allen P, Steadham RE, Vantreese TR, et al. Erectile dysfunction and radiation dose to penile base structures: a lack of correlation. Int J Radiat Oncol Biol Phys 2004; 59: 1039–46. doi: 10.1016/j.ijrobp.2003.12.028 15234037

[10] Tøndel H, Lund Jo-Åsmund, Lydersen S, Wanderås AD, Aksnessæther BY, Jensen CA, et al. Dose to penile bulb is not associated with erectile dysfunction 18 months post radiotherapy: a secondary analysis of a randomized trial. Clin Transl Radiat Oncol 2018; 13: 50–6. doi: 10.1016/j.ctro.2018.09.006 30364704 PMC6198098

[11] Bruner DW, Hunt D, Michalski JM, Bosch WR, Galvin JM, Amin M, et al. Preliminary patient-reported outcomes analysis of 3-dimensional radiation therapy versus intensity-modulated radiation therapy on the high-dose arm of the radiation therapy Oncology group (RTOG) 0126 prostate cancer trial. Cancer 2015; 121: 2422–30. doi: 10.1002/cncr.29362 25847819 PMC4490066

[12] Kundu SD, Roehl KA, Eggener SE, Antenor JAV, Han M, Catalona WJ, et al. Potency, continence and complications in 3,477 consecutive radical retropubic prostatectomies. J Urol 2004; 172(6 Pt 1): 2227–31. doi: 10.1097/01.ju.0000145222.94455.73 15538237

[13] Karsh LI, Gross ET, Pieczonka CM, Aliotta PJ, Skomra CJ, Ponsky LE, et al. Absorbable hydrogel spacer use in prostate radiotherapy: a comprehensive review of phase 3 clinical trial published data. Urology 2018; 115: 39–44. doi: 10.1016/j.urology.2017.11.016 29174940

[14] Naiki T, Okamura T, Nagata D, Mori Y, Kawai N, Ogawa K, et al. Preoperative prediction of neurovascular bundle involvement of localized prostate cancer by combined T2 and diffusion-weighted imaging of magnetic resonance imaging, number of positive biopsy cores, and Gleason score. Asian Pac J Cancer Prev 2011; 12: 909–13. 21790224

[15] Bastasch MD, Teh BS, Mai W-Y, Carpenter LS, Lu HH, Chiu JK, et al. Post-nerve-sparing prostatectomy, dose-escalated intensity-modulated radiotherapy: effect on erectile function. Int J Radiat Oncol Biol Phys 2002; 54: 101–6. doi: 10.1016/S0360-3016(02)02901-2 12182979

[16] Zelefsky MJ, Eid JF. Elucidating the etiology of erectile dysfunction after definitive therapy for prostatic cancer. Int J Radiat Oncol Biol Phys 1998; 40: 129–33. doi: 10.1016/S0360-3016(97)00554-3 9422568

[17] Incrocci L, Slob AK, Levendag PC. Sexual (dys)function after radiotherapy for prostate cancer: a review. Int J Radiat Oncol Biol Phys 2002; 52: 681–93. doi: 10.1016/S0360-3016(01)02727-4 11849790

[18] Cassidy RJ, Yang X, Liu T, Thomas M, Nour SG, Jani AB, et al. Neurovascular bundle-sparing radiotherapy for prostate cancer using MRI-CT registration: a dosimetric feasibility study. Med Dosim 2016; 41: 339–43. doi: 10.1016/j.meddos.2016.08.003 27745996

[19] Cassidy RJ, Nour SG, Liu T, Switchenko JM, Tian S, Ferris MJ, et al. Reproducibility in contouring the neurovascular bundle for prostate cancer radiation therapy. Pract Radiat Oncol 2018; 8: e125–31. doi: 10.1016/j.prro.2017.08.001 28939353 PMC5942224

[20] Ciabatti S, Ntreta M, Buwenge M, Gaudiano C, Sessagesimi E, Romani F, et al. Dominant intraprostatic lesion boosting in sexual-sparing radiotherapy of prostate cancer: a planning feasibility study. Med Dosim 2019; 44: 356–64. doi: 10.1016/j.meddos.2019.01.008 30955990

[21] Wright JL, Newhouse JH, Laguna JL, Vecchio D, Ennis RD. Localization of neurovascular bundles on pelvic CT and evaluation of radiation dose to structures putatively involved in erectile dysfunction after prostate brachytherapy. Int J Radiat Oncol Biol Phys 2004; 59: 426–35. doi: 10.1016/j.ijrobp.2003.11.022 15145159

[22] Samlali H, Udrescu C, Lapierre A, Enachescu C, Ruffion A, Jalade P, et al. Prospective evaluation of a specific technique of sexual function preservation in external beam radiotherapy for prostate cancer. Br J Radiol 2017; 90: 20160877. doi: 10.1259/bjr.20160877 28749171 PMC5853354

[23] Spratt DE, Lee JY, Dess RT, Narayana V, Evans C, Liss A, et al. Vessel-sparing radiotherapy for localized prostate cancer to preserve erectile function: a single-arm phase 2 trial. Eur Urol 2017; 72: 617–24. doi: 10.1016/j.eururo.2017.02.007 28233591

[24] Hamstra DA, Mariados N, Sylvester J, Shah D, Gross E, Hudes R, et al. Sexual quality of life following prostate intensity modulated radiation therapy (IMRT) with a rectal/prostate spacer: secondary analysis of a phase 3 trial. Pract Radiat Oncol 2018; 8: e7–15. doi: 10.1016/j.prro.2017.07.008 28951089

[25] Hötker AM, Mazaheri Y, Zheng J, Moskowitz CS, Berkowitz J, Lantos JE, et al. Prostate cancer: assessing the effects of androgen-deprivation therapy using quantitative diffusion-weighted and dynamic contrast-enhanced MRI. Eur Radiol 2015; 25: 2665–72. doi: 10.1007/s00330-015-3688-1 25820537 PMC4530043

[26] Hwang ME, Mayeda M, Liz M, Goode-Marshall B, Gonzalez L, Elliston CD, et al. Stereotactic body radiotherapy with periprostatic hydrogel spacer for localized prostate cancer: toxicity profile and early oncologic outcomes. Radiat Oncol 2019; 14: 136. doi: 10.1186/s13014-019-1346-5 31375119 PMC6679492

[27] Hannan R, Tumati V, Xie X-J, Cho LC, Kavanagh BD, Brindle J, et al. Stereotactic body radiation therapy for low and intermediate risk prostate cancer-Results from a multi-institutional clinical trial. Eur J Cancer 2016; 59: 142–51. doi: 10.1016/j.ejca.2016.02.014 27035363

[28] Hamstra DA, Shah D, Kurtzman S, Sylvester J, Zimberg SH, Hudes RS, et al. Evaluation of sexual function on a randomized trial of a prostate rectal spacer. J Clin Oncol 2017; 35(6_suppl): 69. doi: 10.1200/JCO.2017.35.6_suppl.69

[29] Jaccard M, Lamanna G, Dubouloz A, Rouzaud M, Miralbell R, Zilli T, et al. Dose optimization and endorectal balloon for internal pudendal arteries sparing in prostate SBRT. Phys Med 2019; 61: 28–32. doi: 10.1016/j.ejmp.2019.04.008 31151576

[30] DiBiase SJ, Wallner K, Tralins K, Sutlief S. Brachytherapy radiation doses to the neurovascular bundles. Int J Radiat Oncol Biol Phys 2000; 46: 1301–7. doi: 10.1016/S0360-3016(99)00551-9 10725644

[31] Merrick GS, Butler WM, Dorsey AT, Lief JH, Donzella JG. A comparison of radiation dose to the neurovascular bundles in men with and without prostate brachytherapy-induced erectile dysfunction. Int J Radiat Oncol Biol Phys 2000; 48: 1069–74. doi: 10.1016/S0360-3016(00)00746-X 11072164

[32] Solan AN, Cesaretti JA, Stone NN, Stock RG. There is no correlation between erectile dysfunction and dose to penile bulb and neurovascular bundles following real-time low-dose-rate prostate brachytherapy. Int J Radiat Oncol Biol Phys 2009; 73: 1468–74. doi: 10.1016/j.ijrobp.2008.06.1946 18922652

[33] Chen LN, Suy S, Uhm S, Oermann EK, Ju AW, Chen V, et al. Stereotactic body radiation therapy (SBRT) for clinically localized prostate cancer: the Georgetown university experience. Radiat Oncol 2013; 8: 58. doi: 10.1186/1748-717X-8-58 23497695 PMC3610192

[34] Obayomi-Davies O, Chen LN, Bhagat A, Wright HC, Uhm S, Kim JS, et al. Potency preservation following stereotactic body radiation therapy for prostate cancer. Radiat Oncol 2013; 8: 256. doi: 10.1186/1748-717X-8-256 24180317 PMC4228383

[35] Kishan AU, Park SJ, King CR, Roberts K, Kupelian PA, Steinberg ML, et al. Dosimetric benefits of hemigland stereotactic body radiotherapy for prostate cancer: implications for focal therapy. Br J Radiol 2015; 88: 20150658. doi: 10.1259/bjr.20150658 26463234 PMC4984947

[36] Loi M, Wortel RC, Francolini G, Incrocci L. Sexual function in patients treated with stereotactic radiotherapy for prostate cancer: a systematic review of the current evidence. J Sex Med 2019; 16: 1409–20. doi: 10.1016/j.jsxm.2019.05.019 31303575

[37] Dess RT, Hartman HE, Aghdam N, Jackson WC, Soni PD, Abugharib AE, et al. Erectile function after stereotactic body radiotherapy for localized prostate cancer. BJU Int 2018; 121: 61–8. doi: 10.1111/bju.13962 28710895

[38] Fuller DB, Naitoh J, Mardirossian G. Virtual HDR CyberKnife SBRT for localized prostatic carcinoma: 5-year disease-free survival and toxicity observations. Front Oncol 2014; 4: 321. doi: 10.3389/fonc.2014.00321 25505732 PMC4241836

[39] Madsen BL, Hsi RA, Pham HT, Presser J, Esagui L, Corman J, et al. Intrafractional stability of the prostate using a stereotactic radiotherapy technique. Int J Radiat Oncol Biol Phys 2003; 57: 1285–91. doi: 10.1016/S0360-3016(03)00746-6 14630263

[40] Liss A, Zhou J, Evans C, Murgic J, Narayana V, Hamstra DA, et al. Anatomic variability of the neurovascular elements defined by MRI. Brachytherapy 2014; 13: S42–3. doi: 10.1016/j.brachy.2014.02.267

[41] Hong H, Koch MO, Foster RS, Bihrle R, Gardner TA, Fyffe J, et al. Anatomic distribution of periprostatic adipose tissue: a mapping study of 100 radical prostatectomy specimens. Cancer 2003; 97: 1639–43. doi: 10.1002/cncr.11231 12655520

