# Supplementary material for: Dosimetric feasibility of neurovascular bundle-sparing stereotactic body radiotherapy with periprostatic hydrogel spacer for localized prostate cancer to preserve erectile function
Source: Br J Radiol. 2021 Mar 2;94(1119):20200433. doi: 10.1259/bjr.20200433 (PMC8011244; doi:10.1259/bjr.20200433)
Supplement: Supplementary Material 1. [file bjr.20200433.suppl-01.docx]

**Supplementary Table:** Detailed MRI protocol parameters

| Series | pulse sequence | FOV | Thickness | Gap | Frequency | Phase | TR | TE | Flip Angle | ETL | NEX | Bandwidth |
| --- | --- | --- | --- | --- | --- | --- | --- | --- | --- | --- | --- | --- |
| Localizer | Spin echo | 48 | 8.0 | 3 | 256 | 160 | min | Min | N/A | N/A | N/A | N/A |
| Calibration | Gradient echo | 48 | 15.0 | 0 | N/A | N/A | N/A | N/A | N/A | N/A | N/A | N/A |
| Sagittal T2 | SSFSE | 30 | 4.5 | 0 | 256 | 192 | 2500 | 80 | N/A | N/A | N/A | 83 |
| Axial FSE T2 | FSE | 18 | 3.0 | 0 | 320 | 224 | 9127 | 120 | 160 | 25 | 4 | 50 |
| Axial T1 | FSE | 18 | 3.0 | 0 | 320 | 192 | 700 | MIN FULL | 111 | 4 | 2 | 50 |
| Axial DWI 50, 1000 | Spin echo | 22 | 3.0 | 0 | 80 | 96 | 7000 | Min | N/A | N/A | 2,16 | 250 |
| Axial DWI 50, 1500 | Spin echo | 22 | 3.0 | 0 | 80 | 96 | 7000 | Min | N/A | N/A | 2,16 | 250 |
| Coronal T2 | FSE | 18 | 3.0 | 0 | 320 | 224 | 9127 | 120 | 160 | 25 | 4 | 50 |
| Axial T1 Fat Sat | LAVA-Flex | 32 | 4.0 | 0 | 320 | 160 | N/A | MIN FULL | 12 | N/A | 1 | 166 |
| Axial T1 dynamic | Vasc TOF SPGR | 32 | 4.0 | 0 | 128 | 128 | 2 | Min | 10 | N/A | 1 | 62 |
| Axial T1 post | LAVA-Flex | 32 | 4.0 | 0 | 320 | 160 | N/A | MIN FULL | 12 | N/A | 1 | 166 |
